# The effectiveness of long-needle acupuncture at acupoints BL30 and BL35 for CP/CPPS: a randomized controlled pilot study

**DOI:** 10.1186/s12906-017-1768-2

**Published:** 2017-05-12

**Authors:** Minjie Zhou, Mingyue Yang, Lei Chen, Chao Yu, Wei Zhang, Jun Ji, Chi Chen, Xueyong Shen, Jian Ying

**Affiliations:** 1 Shanghai Qigong Research Institute, Shanghai, China; 2 Graduate School, The Shanghai University of Traditional Chinese Medicine, Shanghai, China; 3 The Department of Pain Management, Luoyang Central Hospital, Luoyang, China; 4 The Longhua Hospital Shanghai University of Traditional Chinese Medicine, Shanghai, China; 5 Shanghai TCM-INTEGRATED Hospital, Shanghai, Shanghai University of Traditional Chinese Medicine, Shanghai, China

**Keywords:** BL30, BL35, CP/CPPS, Acupuncture, Long needle

## Abstract

**Background:**

The chronic prostatitis/chronic pelvic pain syndrome (CP/CPPS) is one of the commonest chronic inflammatory diseases in adult men, for which acupuncture has been used to relieve related symptoms. The present study aimed to evaluate the therapeutic effect of the long-needle acupuncture on CP/CPPS.

**Methods:**

A randomized traditional acupuncture-controlled single blind study was conducted on 77 patients who were randomized into long-needle acupuncture (LA) and traditional acupuncture (TA) groups. The patients received six sessions of acupuncture for 2 weeks and a follow-up was scheduled at week 24. The primary outcome was measured by the total National Institutes of Health-Chronic Prostatitis Symptom Index (NIH-CPSI) score at week 2. Four domains of the NIH-CPSI (urination, pain or discomfort, effects of symptoms, and quality of life) and the clinical efficacy score served as the secondary outcome.

**Results:**

The total NIH-CPSI score at week 2 and week 24 was significantly improved in the LA group compared with the TA group. LA significantly improved urination, pain or discomfort, the effects of symptoms, and the quality of life at week 2 and week 24 and patients undergoing LA treatment had a higher clinical efficacy score.

**Conclusion:**

Needling at the BL30 and BL35 using LA benefits patients with CP/CPPS.

**Trial registration:**

The study was registered at the Chinese Clinical Trial Register (ChiCTR-ICR-15006138).

## Background

The chronic prostatitis/chronic pelvic pain syndrome (CP/CPPS) is one of the commonest chronic inflammatory diseases in male adults, the prevalence of which is estimated to range from 2% to 10% and the overall lifetime prevalence from 9% to 16% [1]. The symptoms of CP/CPPS manifest as pelvic pain, prostate inflammation, voiding symptoms, and sexual disturbance [1, 2], which differ from the voiding problem in benign prostatic hyperplasia, another common prostatic problem in male adults [3]. Pain has a significant effect on the quality of life in patients with CP/CPPS. Antibiotics are the most common treatment for patients with CP/CPPS, but the antibiotic regimen remains controversial in clinical practice and the number of prostatic bacteria in patients with CP/CPPS does not differ significantly from that in asymptomatic patients [4]. Fewer than 10% of patients with CP/CPPS are found to have bacteria in their urinary tracts [1], and systematic reviews have shown no sufficient evidence to support the benefits of antibiotics for patients with CP/CPPS [5, 6]. Collective evidence has shown that CP/CPPS is an inflammatory disease [7], for which anti-inflammatory medications are commonly prescribed. However, no large-scale randomized control trials have been conducted to demonstrate the effectiveness of anti-inflammatory medication in these patients [5, 6] and their adverse effects in elderly patients, such as gastrointestinal disturbance, are a serious concern [4, 7, 8]. Current evidence has not supported the benefits of the commonly used medication, alpha-blockers, in CP/CPPS patients [5, 6] and the unsatisfactory nature of conventional medications leads patients to seek alternative therapies.

Acupuncture has been used to treat CP/CPPS in a few clinical studies [9]. A meta-analysis indicated that acupuncture results in statistical and clinical improvements in the voiding domain of the National Institutes of Health-Chronic Prostatitis Symptom Index (NIH-CPSI) in CP/CPPS patients [10]. The observational studies and randomized controlled trials revealed that acupuncture also reduced pain and improved the immune function in patients with CP/CPPS [11–14]. The efficacy of acupuncture is determined by the various parameters, e.g. the depth of the needles, the number of acupoints, and needle manipulation [15, 16], which need to be optimized to maximize its effectiveness.

In clinical practice, long-needle treatment effectively produces the Deqi sensation in the deep pelvic area and induces the constriction of the deep transverse perineal muscle, which is thought to be effective for CP/CPPS. Previous observational studies revealed that deep needling at BL30 at a depth of 3.5–4.5 cun significantly improved the clinical symptoms of CP [17], as did needling at BL35 at a depth of 2–3 cun [18]. The traditional filiform needle hardly produces the Deqi sensation in the deep pelvic area. The present investigation aimed to evaluate effectiveness of LA at acupoints BL30 and BL35 in CP compared with traditional acupuncture (TA) in a single blind randomized pilot study.

## Methods

### Setting and study design

A randomized traditional acupuncture-controlled single blind trial was conducted. The study protocol was approved by Long Hua Hospital Medical Ethics Committee, Shanghai University of Traditional Chinese Medicine (2014LCSY31). The random codes were generated by SPSS 16.0., sealed in opaque envelopes in sequence, and kept by the principal investigator (JY). Patients were recruited from the Long Hua Hospital Shanghai University of Traditional Chinese Medicine and the clinics of the Shanghai Qigong Research Institute. Eligible patients received treatment with either long-needle or traditional acupuncture for 2 weeks. The evaluation of efficacy was performed individually by the assessor, who was blind to the type of acupuncture intervention. The acupuncturist was not involved in the evaluation of efficacy, or data processing and analysis.

### Subjects

#### Sample size

The sample size was determined by the significant difference between LA group and TA group with an effect size of 0.7 for a two-sided significance level of 5% (two-tailed) and 0.8 power. Each group required 34 patients. We estimated a 10% drop-out rate and the sample size was set at 76 (38 per group). In the study, the last two patients were recruited from two study sites at the same time, thus a total of 77 patients were recruited.

#### Inclusion criteria

The patients included (1) were diagnosed with CP/CPPS according to *Urology* (edited by Dr. Jieping Wu) [19];,(2) had the symptoms for more than 3 months, (3) were men aged 20–50 years, and (4) were able to read, understand, and sign the informed consent form.

#### Exclusion criteria

Patients that were excluded (1) had acute prostatitis, (2) had a prostate tumor, (3) had primary benign prostatic hyperplasia,(4)had urinary tract infections or urethritis, (5) had urinary stones, epididymitis, groin hernia, varicocele, or pelvic pain caused by the colorectal or lumbar diseases, (6) had severe heart, liver, kidney, or hematopoietic problems, psychiatric disorders, or other life-threatening diseases, and (7) were unable to complete all of the treatments.

### Intervention

#### LA treatment

The acupuncture treatment in both groups was performed by licensed acupuncturists with more than 3 years of clinical experience. The patient was placed in the prone position on the acupuncture bed. The bilateral BL30 (Baihuan Shu) and BL35 (Huiyang) acupoints were used for the treatments. BL30 is at 1.5 cun lateral to the midline of the sacral crest and at the level of the fourth posterior sacral foramen. BL35 is at 0.5 cun lateral to the midline and at the level with the tip of the coccyx. The long needle (0.4 × 100 mm) was inserted perpendicularly at BL30 (bilateral) at a depth of 75–90 mm (3 ~ 3.5 cun). The Deqi sensation (the feeling of soreness, numbness, distension, and heaviness) from the local site to the perineum (including the urethra and anus) was achieved by the twirling and lifting-thrusting of the needle. In addition, the long needle (0.4 × 100 mm) was inserted obliquely at BL35 (bilateral) with the pin of the needle pointing to the ischial rectal fossa at a depth of 3 ~ 3.5 cun. The Deqi sensation from the local site to perineum was achieved. Electrical stimulation was applied to the acupoints with the paired ipsilateral BL30 (the anode) and BL35 (the cathode) under a continuous wave of 2.5-Hz frequency current for 30 min (Yindi KWD-808-I acupuncture device, Jiangsu, China). The intensity of the electrical current was determined by the tolerance of the patients. The treatment was performed three times per week for 2 weeks (six sessions).

#### TA treatment

Patients received the same procedures for 2 weeks but traditional treatment was applied. Briefly, the traditional filiform needle (0.3 × 40 mm) was inserted perpendicularly into the bilateral BL30 and BL35 at a depth of 25–35 mm. The Deqi sensation was induced at local site only. The intensity of electrical stimulation (2.5-Hz, continuous wave for 30 min) was the same as that received by the treatment group.

### Follow-up

A follow-up was scheduled at week 24 after the patients had started the intervention in our study. The assessor evaluated patients over the telephone during both working hours and non-working hours. The patient was defined as lost to follow-up if he could not be contacted by the assessor within 1 week.

### Outcome

The NIH-CPSI was used as the primary outcome measurement. Four domains of NIH-CPSI, including pain or discomfort, urination, the effects of symptoms, and quality of life, were evaluated in a secondary analysis.

The clinical efficacy as the secondary outcome was assessed by determining the percentage of the change in the NIH-CPSI score after treatment ((*score after treatment* – *score before treatment*)/*score before treatment* × 100%). A 30% of improvement in NIH-CPSI score was considered clinically effective.

The NIH-CPSI assessment was performed at baseline, week 2, and week 24 (follow-up).

### Data analysis

SPSS 16.0 was used for data analysis with the intention-to-treat approach. The last observation carried forward approach was used to estimate the missing value. The *t*-test was used for continuous data between two groups or time-points. The Chi-square test was used for evaluating clinical efficacy. All results were reported as the mean ± standard deviation (SD). A *P* value of less than 0.05 was considered to be statistically significant.

## Results

**Fig. 1:**
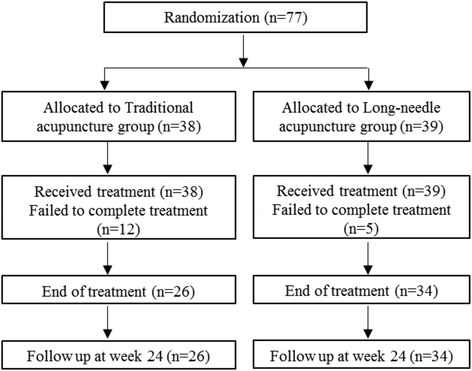
Flow diagram of patient recruitment and follow-up

Seventy-seven patients were recruited in the study, 39 of whom received the long-needle treatment and 38 traditional acupuncture treatment. The flowchart is shown in Fig. 1.

The baseline characteristics of the two groups with regard to age, disease duration, NIH-CPSI score and its domains (pain or discomfort score, urination, effects of symptoms, and quality of life), and Chinese Medicine symptom score showed no significant difference (shown in Table 1).

**Table 1:** Baseline characteristics of patients $\left(\overline{\upchi}\pm \mathrm{S}\right)$

Demographic information	Control group (*n* = 38)		Treatment group (*n* = 39)		*P* value
	Mean ± SD	Range	Mean ± SD	Range	
Age (year)	31.7 ± 6.2	21–44	31.4 ± 7.0	24–50	0.84
Duration (month)	32.4 ± 39.3	3–156	24.3 ± 27.9	3–120	0.31
NIH-CPSI (total)	26.7 ± 4.9	17–37	26.6 ± 5.8	12–37	0.89
Pain or discomfort	11.1 ± 2.8	5–17	11.1 ± 3.1	4–16	1.0
Urination	5.8 ± 2.8	0–10	5.4 ± 3.3	0–11	0.52
Impact of symptoms	5.0 ± 1.0	2–6	5.2 ± 1.1	3–6	0.34
Quality of life	4.9 ± 1.2	2–6	4.9 ± 1.1	3–6	0.84
CM symptom	15.3 ± 7.3	6–34	14.0 ± 5.0	6–24	0.36

### Treatment with LA improved the NIH-CPSI scores

After 2 weeks of acupuncture treatment, the total score of the NIH-CPSI and its four domains (pain or discomfort, urination, effects of symptoms, and quality of life) were significantly decreased in patients who received either LA or TA (*P* < 0.05) (Table 2). In the LA group, the total score and its four domains were significantly lower than those in the TA group (*P* < 0.05). A 22-week follow-up indicated that the LA treatments reduced the total score and three of its four domains (pain or discomfort, effects of symptoms, and quality of life, but not the urination domain) significantly compared with TA (*P* < 0.05).

**Table 2:** NIH-CPSI scores and Chinese Medicine (CM) symptoms after the intervention $\left(\overline{\upchi}\pm \mathrm{S}\right)$

Scores	TA (*n* = 38)			LA (*n* = 39)		
	Pre-treatment	Post-treatment	Follow up	Pre-treatment	Post-treatment	Follow up
NIH-CPSI (total)	26.7 ± 4.9	22.6 ± 5.2*	17.3 ± 9.1*	26.6 ± 5.8	17.0 ± 6.0*^△^	9.4 ± 8.6*^△^
Pain or discomfort	11.1 ± 2.8	9.0 ± 2.7*	6.6 ± 4.0*	11.1 ± 3.1	6.5 ± 3.0*^△^	3.3 ± 3.8*^△^
Urination	5.8 ± 2.8	5.0 ± 2.0*	3.8 ± 3.1*	5.4 ± 3.3	3.5 ± 2.6*^△^	2.1 ± 2.6*
Impact of symptoms	5.0 ± 1.0	4.4 ± 1.2*	3.4 ± 1.8*	5.2 ± 1.1	3.5 ± 1.6*^△^	2.0 ± 1.7*^△^
Quality of life	4.9 ± 1.2	4.4 ± 1.4*	3.5 ± 1.7*	4.9 ± 1.1	3.5 ± 1.3*^△^	2.0 ± 1.6*^△^
CM symptom	15.3 ± 7.3	12.1 ± 5.7*	-	14.0 ± 5.0	8.4 ± 5.3*^△^	-

*, *P* < 0.05 when comparison the pre-treatment score and post-treatment score in the same group; △, *P* < 0.05 when comparing the long-needle acupuncture group and the conventional acupuncture group. An ITT method was applied for analysis

### LA group had greater improvement in clinical efficacy

After 2 weeks of acupuncture treatment, the Chinese Medicine symptoms in both groups were significantly improved compared with the baselines while the long-needle acupuncture group had a greater reduction from the baseline compared with the TA group (Table 2, CM symptoms). LA had significantly higher clinical effectiveness (30% improvement of NIH-CPSI score) compared to TA (*P* = 0.001) as shown in Table 3.

**Table 3:** Clinical efficacy in the long-needle acupuncture and traditional acupuncture groups at week 2 (*n* = 77)

	TA	LA	
	(*n* = 38)	(*n* = 39)	*p*-value
Clinical efficacy, No. (%)			
Effective	8 (21.1)	22 (56.4)	0.001
Ineffective	30 (78.9)	17 (43.6)	

*LA* long-needle acupuncture; *TA* traditional acupuncture; ITT method and chi-square test was used for the analysis

## Discussion

CP/CPPS is the term used in western medicine, but its symptoms were described in ancient books of traditional Chinese medicine as, e.g., the sperm-turbid, white-turbid, and red-turbid. The clinical symptoms of CP are mainly demonstrated in two ways: one is pain in the pelvic cavity and discomfort in other parts and the other is abnormal urination. TA treatment for CP involves choosing main acupoints in the lower abdomen and lumbosacral region [4], such as RN3 (Zhongji), RN4 (Guanyuan), SP6 (Sanyinjiao), and L23 (Shenshu).

Our previous studies showed that needling at the bilateral BL30 and BL35 significantly improved CP [20, 21]. In Chinese Medicine doctrine, BL30 is an acupoint corresponding to internal essence, where the essence Qi of the human body infuses. This acupoint has the effect of dredging meridians and collaterals, and supplementing the kidney and essence. BL35 was characterized as having the effect of invigorating Qi and removing wet. Acupuncture at the two acupoints could clear dampness-heat, promote blood circulation, and invigorate Qi for the human body. The scientific action mechanism of the electrical deep needling in the study remains largely unexplored. It may be partially associated with the pelvic floor stimulation, which has been used as a physiotherapy to relieve the chronic pelvic pain and incontinence in patients [22–24].

In previous studies, we applied LA with electrical stimulation bilaterally at BL30 and BL35 for CP concurrently with Chinese herbal medicine [20, 21]. We found that needling at BL30 and BL35 could enhance the pudendal nerve stimulation, whereby the concurrent use of acupuncture and Chinese herbal medicine significantly benefited patients with CP/CPPS compared with treatment with Chinese herbal medicine only. In the present study, we examined whether needling at BL30 and BL35 had a therapeutic effect compared with the TA treatment. The findings demonstrated that LA had a superior effect than TA, although the change in the absolute value of the NIH-CPSI score was small but significant. For example, the difference in the NIH-CPSI score was 4.7 (4.7/43*100% = 11.9%). Because the control in the study was a positive treatment, the difference between the two treatments is relatively smaller than that which a sham control would provide. However, it pragmatically reflects real clinical practice and evaluates the effectiveness of LA treatment rather than its efficacy. In fact, the change in NIH-CPSI from baseline was 24.1% after long-needle treatment. The clinical efficacy analysis (30% of improvement in NIH-CPSI score) indicated LA is clinically superior to TA.

In the present study, both TA and LA needling at BL30 and BL35 had a therapeutic effect on CP/CPPS. However, when needling at the same acupoints, the deep insertion of the long needle significantly improved CP/CPPS compared with TA. The stimulation of the pudendal nerve by needles may be the key mechanism to the relief of CP/CPPS. The pudendal nerve is the main sensory and motor nerve of the perineum and supplies sensation from the genitalia and control of the urethral sphincter and anal sphincter. Anatomically, the pudendal nerve passes through the tissue under the deep area at BL30 and BL35; therefore, the deep insertion of long needles at these acupoints may enhance the stimulation of the pudendal nerve and thereby promote the inhibition of local sensations passing to the central nervous system [25, 26]. The long-needle manipulation may trigger a stronger contraction of the deep transverse perineal muscle, and consequently improve the local blood circulation of prostate and reduce inflammation in the pelvis [17, 27]. The mechanism needs to be further determined.

In this study, no serious adverse effects were found. Skin bruising and slight bleeding were occasionally observed in both groups and deep needling increases the risk of pelvic injuries. The long-needle treatment should be performed by an experienced acupuncturist. Urinalysis and a fecal occult blood test are recommended to monitor any potential injuries.

The study had limitations. The sample size in the pilot study was small. A large number of patients didn’t complete the intervention in the TA and LA groups. One of the reasons for dropping out was that the patients were not tolerant to acupuncture treatment. The majority of reasons for dropping out were not detected as most patients could not be reached by telephone. The study had only one time-point of follow-up while follow-ups need to be made at multiple time-points in the future. Objective parameters, e.g. white blood cells in expressed prostatic secretion, could be used to evaluate chronic prostatitis. As the present study evaluated the effectiveness of acupuncture, the potential placebo effect cannot be excluded. A sham acupuncture can be used to measure the efficacy of acupuncture by excluding the potential placebo effect.

## Conclusion

The present study demonstrated that deep needling at BL 30 and BL35 using LA significantly improved CP/CPPS compared with TA. The study supported a large-scale randomized controlled trial to evaluate LA for CP/CPPS.

## References

[1] Strauss AC, Dimitrakov JD (2010). New treatments for chronic prostatitis/chronic pelvic pain syndrome. Nat Rev Urol.

[2] Kwon JK, Chang IH (2013). Pain, catastrophizing, and depression in chronic prostatitis/chronic pelvic pain syndrome. Int Neurourol J.

[3] Nickel JC (2008). Inflammation and benign prostatic hyperplasia. Urol Clin North Am.

[4] Pontari MA (2003). Chronic prostatitis/chronic pelvic pain syndrome in elderly men: toward better understanding and treatment. Drugs Aging.

[5] Le B, Schaeffer AJ. Chronic prostatitis. BMJ Clin Evid. 2011;2011 PMC3275320 21736764

[6] McNaughton C, Mac Donald R, Wilt T (2001). Interventions for chronic abacterial prostatitis. Cochrane Database Syst Rev.

[7] Duclos AJ, Lee CT, Shoskes DA (2007). Current treatment options in the management of chronic prostatitis. Ther Clin Risk Manag.

[8] Udoji MA, Ness TJ (2013). New directions in the treatment of pelvic pain. Pain Manag.

[9] Lee SH, Lee BC (2011). Use of acupuncture as a treatment method for chronic prostatitis/chronic pelvic pain syndromes. Current urology reports.

[10] Cohen JM, Fagin AP, Hariton E, Niska JR, Pierce MW, Kuriyama A, Whelan JS, Jackson JL, Dimitrakoff JD (2012). Therapeutic intervention for chronic prostatitis/chronic pelvic pain syndrome (CP/CPPS): a systematic review and meta-analysis. PLoS One.

[11] Lee SH, Lee BC (2009). Electroacupuncture relieves pain in men with chronic prostatitis/chronic pelvic pain syndrome: three-arm randomized trial. Urology.

[12] Lee SW, Liong ML, Yuen KH, Krieger JN (2014). Acupuncture and immune function in chronic prostatitis/chronic pelvic pain syndrome: a randomized, controlled study. Complement Ther Med.

[13] Lee SW, Liong ML, Yuen KH, Leong WS, Chee C, Cheah PY, Choong WP, Wu Y, Khan N, Choong WL *et al*: Acupuncture versus sham acupuncture for chronic prostatitis/chronic pelvic pain. *Am J Med* 2008, 121(1):79 e71–77. 10.1016/j.amjmed.2007.07.033 PMC4497585 18187077

[14] Tugcu V, Tas S, Eren G, Bedirhan B, Karadag S, Tasci A (2010). Effectiveness of acupuncture in patients with category IIIB chronic pelvic pain syndrome: a report of 97 patients. Pain Med.

[15] Carlsson C (2002). Acupuncture mechanisms for clinically relevant long-term effects--reconsideration and a hypothesis. Acupunct Med.

[16] MacPherson H, Altman DG, Hammerschlag R, Youping L, Taixiang W, White A, Moher D, Group SR (2010). Revised STandards for reporting interventions in clinical trials of acupuncture (STRICTA): extending the CONSORT statement. PLoS Med.

[17] Ge J, Ge S (2001). Clinical observation on treatment of chronic prostatitis with deeply needling main point Baihuanshu. Chinese Acuponcture & Moxibustion.

[18] Zhong W, Yuan Y (2003). Acupunture on Huiyang acupoint (BL35) for chronic prostatitis: a clinical observation of 30 cases. Journal of Clinical Acupuncture and Moxibustion.

[19] Wu J (2004). WU Jieping Urology.

[20] Yang MY, Ying J, Li JX, Wang SY, Zhou MJ, Zhang W (2014). Clinical observation of Electroacupuncture at Baihuanshu(BL30) and Huiyang(BL35) for chronic prostatitis. Shanghai Journal of Acupuncture and Moxibustion.

[21] Ying J, Li JX, Wang SY, Yue YM (2014). Zhou Mj, Shang YY: **clinical observation of Electroacupuncture at Baihuanshu(BL30) and Huiyang(BL35) for chronic Abacterial prostatitis**. Shanghai Journal of Acupuncture and Moxibustion.

[22] Goode PS, Burgio KL, Johnson TM, Clay OJ, Roth DL, Markland AD, Burkhardt JH, Issa MM, Lloyd LK (2011). Behavioral therapy with or without biofeedback and pelvic floor electrical stimulation for persistent postprostatectomy incontinence: a randomized controlled trial. JAMA.

[23] Doggweiler R, Stewart AF (2011). Pelvic floor therapies in chronic pelvic pain syndrome. Current urology reports.

[24] Polackwich AS, Li J, Shoskes DA (2015). Patients with pelvic floor muscle spasm have a superior response to pelvic floor physical therapy at specialized centers. J Urol.

[25] Zheng H (1993). The deep needling at BL29 and BL35 for voiding dysfunctions. Shanghai Journal of Acupuncture and Moxibustion.

[26] Chen Y, Shen P, Chen G, Zhang L (2001). Experimental study on the effect of Electroacupuncture of "Huiyang" and "Zhonglushu" on Urodynamics in nonbacterial prostatitis rats. Acupuncture Research.

[27] Wang s-y, Chen G-m, Li L-h: "Four sacral needles" therapy for female stress incontinence. Shanghai Journal of Acupuncture and Moxibustion 2006, 25(5):13–15.

